# Letter in response to the case report: “Recalcitrant generalized granuloma annulare treated successfully with dupilumab”

**DOI:** 10.1016/j.jdcr.2023.07.018

**Published:** 2023-07-26

**Authors:** Claudia Paganini, Marina Talamonti, Elena Campione, Luca Bianchi, Marco Galluzzo

**Affiliations:** a Department of Systems Medicine, University of Rome “Tor Vergata”, Rome, Italy; b Dermatology Unit, Fondazione Policlinico Tor Vergata, Rome, Italy

**Keywords:** dupilumab, generalized granuloma annulare, granuloma annulare, recalcitrant granuloma annulare

*To the Editor:* We are grateful to share our experience with you concerning a generalized form of granuloma annulare (GA) treated with dupilumab off-label. Following your case report^1^ and based on the article published by Min et al,^2^ which emerged that an inflammatory component of Th2 types is also present in GA, we decided to treat our patient with dupilumab.

We report the case of an 81-years-old woman affected by a generalized form of GA that appeared 3 years prior, initially localized on the upper limbs and then extending to the entire trunk and a portion of the lower limbs (Fig 1). The diagnosis was histologically confirmed by a pathologist in 2019 (Fig 2). Anamnesis was negative for atopic dermatitis and allergic comorbidities.

**Fig 1:**
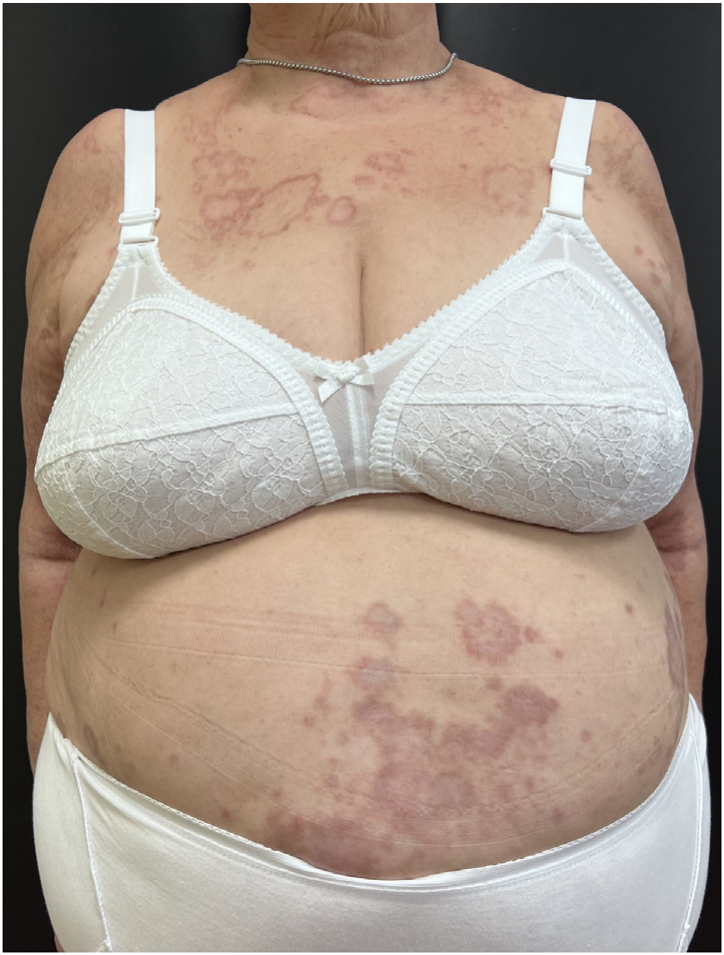
Patient at baseline: granuloma annulare on the trunk and *upper* limbs.

**Fig 2:**
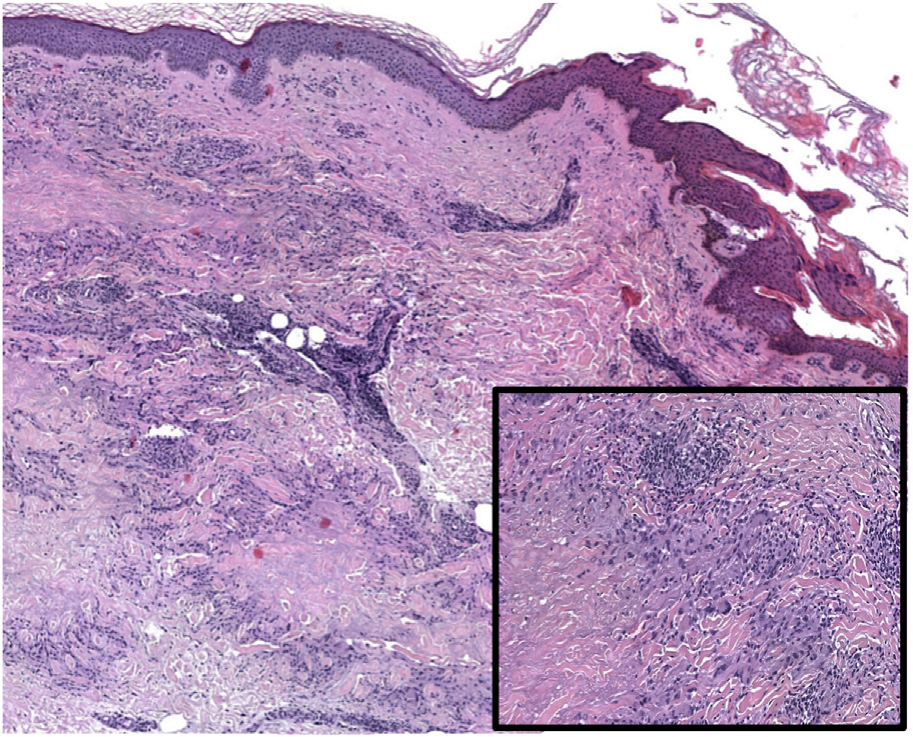
Histopathology image of granuloma annulare: granulomatous inflammatory pattern situated within the superficial and mid dermis; the dermis in granuloma annulare reveals histiocytes arranged in an interstitial pattern, the presence of multinucleate giant cells, and a mild perivascular lymphocytic infiltrate.

After several therapy failures such as infliximab, doxycycline, and methotrexate, dupilumab was administered at a loading dose of 600 mg at baseline and subsequently 300 mg every 2 weeks [Figs 3, *A* and 4, *A*].

**Fig 3:**
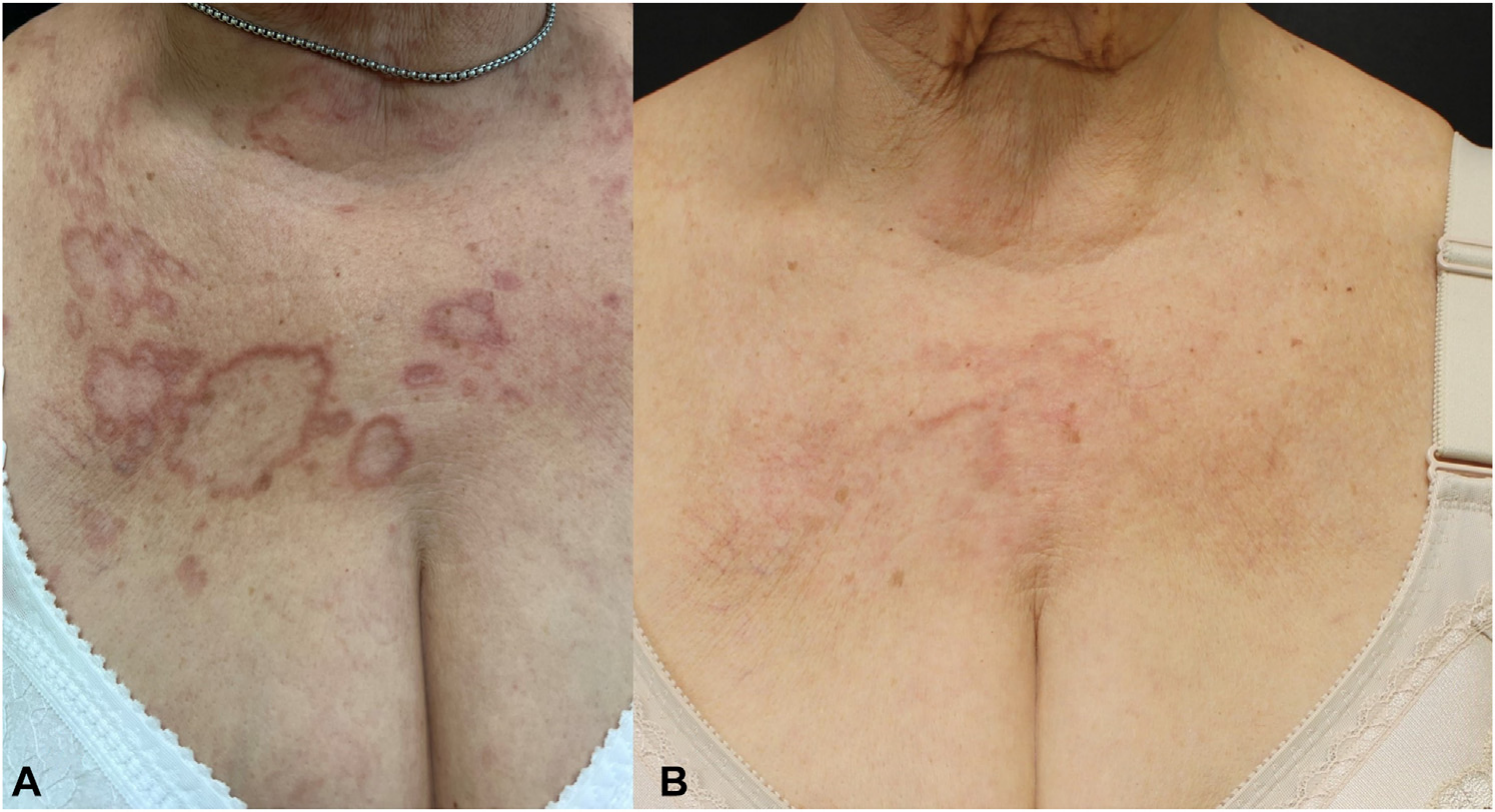
**A,** A particular of *upper* chest at baseline. **B,** A particular of *upper* chest after 24 weeks of dupilumab.

**Fig 4:**
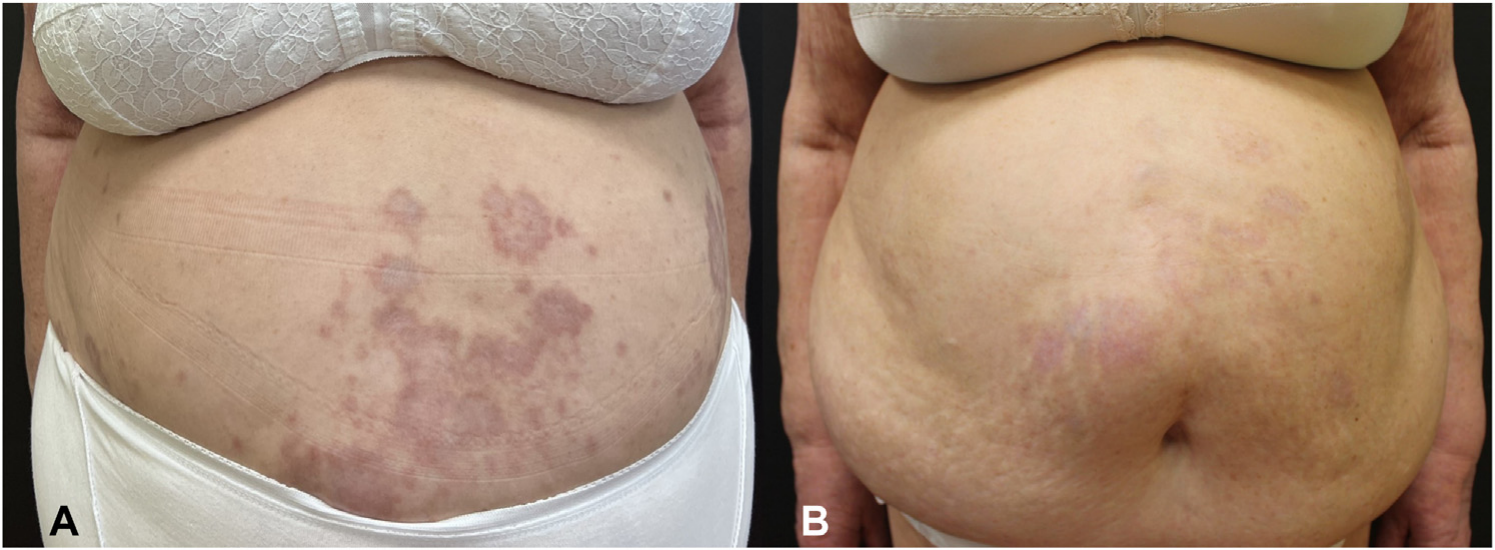
**A,** Patient’s abdomen at baseline. **B,** Patient’s abdomen after 24 weeks of dupilumab.

At her 4-weeks follow-up, the lesions had become less erythematous and infiltrated. At her 16-weeks follow-up, the signs of inflammation nearly disappeared, with the lesions resolving and the presence of postinflammatory hyperpigmentation. At her 24-weeks follow-up, we achieved the resolution of most of the lesions with some hyperpigmentation (Figs 3, *B* and 4, *B*).

GA is a chronic inflammatory, noninfectious granulomatous skin disease with an unknown etiology. Localized GA typically resolves on its own, while the generalized form, which accounts for approximately 15% of cases,^3^ can be more resistant and challenging to treat.

Morphological similarities to other forms of granuloma suggest that GA is caused by a Th1 inflammatory reaction, leading to the use of drugs that inhibit this Th1 activation, such as tumor necrosis factor-alpha inhibitors.^4^ Despite best efforts, therapy often fails, suggesting that an alternative pathway may be involved in the development of GA, as in the case presented. The response to Th1 or Th2 inhibition may depend on the stage of granuloma formation.^1^ In a case with a long-standing GA history unresponsive to tumor necrosis factor-alpha inhibitors, Th2 signaling likely predominated, explaining the positive response to dupilumab.

Moreover, according to the article by Min et al,^2^ it is evident that in cases where the patient lacks atopic comorbidities, there is still an upregulation of Th2-related markers in nonlesional skin affected by GA, indicating the presence of ongoing systemic inflammation. These findings are consistent with similar observations in other inflammatory skin disorders, emphasizing the necessity for clinical trials utilizing systemic agents to specifically target systemic inflammation and effectively manage generalized GA.^2^

In conclusion, through this letter, we aim to emphasize the significance of Th2 skewing in GA, characterized by pronounced upregulation of interleukin 4 and elevation of JAK3. These observations point toward a potential role for targeted treatments such as dupilumab, which may soon be recognized as a valuable addition to the armamentarium for managing refractory forms of GA.

## Conflicts of interest

None disclosed.

## References

[1] Eingun J.S., Joshua B., Sterling F. (2020). Recalcitrant generalized granuloma annulare treated successfully with dupilumab. JAAD Case Rep.

[2] Min M.S., Wu J., He H. (2020). Granuloma annulare skin profile shows activation of T-helper cell type 1, T-helper cell type 2, and Janus kinase pathways. J Am Acad Dermatol.

[3] Wang J., Khachemoune A. (2018). Granuloma annulare: a focused review of therapeutic options. Am J Clin Dermatol.

[4] Min M.S., Lebwohl M. (2016). Treatment of recalcitrant granuloma annulare (GA) with adalimumab: a single-center, observational study. J Am Acad Dermatol.

